# Prognostic Role of BRAF Mutation in Stage II/III Colorectal Cancer Receiving Curative Resection and Adjuvant Chemotherapy: A Meta-Analysis Based on Randomized Clinical Trials

**DOI:** 10.1371/journal.pone.0154795

**Published:** 2016-05-03

**Authors:** Lizhen Zhu, Caixia Dong, Ying Cao, Xuefeng Fang, Chenhan Zhong, Dan Li, Ying Yuan

**Affiliations:** 1 Department of Medical Oncology, The Second Affiliated Hospital of Zhejiang University School of Medicine, Hangzhou, Zhejiang Province, China; 2 Cancer Institute, Key Laboratory of Cancer Prevention and Intervention, Chinese National Ministry of Education, Key Laboratory of Molecular Biology in Medical Sciences, The Second Affiliated Hospital of Zhejiang University School of Medicine, Hangzhou, Zhejiang Province, China; University of Queensland Diamantina Institute, AUSTRALIA

## Abstract

**Background and Objective:**

Studies examining the prognostic value of the BRAF mutation on relapse-free survival (RFS), disease-free survival (DFS) and overall survival (OS) in stage II/III colorectal cancer (CRC) patients receiving curative resection and adjuvant chemotherapy so far showed discrepant results. Therefore, a meta-analysis of relevant studies was performed for clarification.

**Methods:**

Randomized trials of stage II/III colorectal cancer treated with curative resection followed by adjuvant chemotherapy were selected to conduct a meta-analysis. The necessary descriptive and statistical information such as hazard ratios (HRs) and 95% confidence intervals (CIs) were derived from published survival data.

**Results:**

Seven phase III randomized clinical trials (RCTs) including 1,035 BRAF mutation stage II/III CRC patients receiving curative resection and adjuvant chemotherapy were analyzed. Overall, BRAF mutation resulted in poorer OS (HR = 1.42, 95% CI: 1.25–1.60; P < 0.00001), and poorer DFS (HR = 1.26, 95% CI: 1.07–1.48, P = 0.006) compared with BRAF wild-type CRC. The prognostic role on RFS could not be elucidated in the meta-analysis because of limited data.

**Conclusions:**

BRAF mutation was significantly related with shorter DFS and OS among stage II/III CRC patients receiving adjuvant chemotherapy after curative resection. Its prognostic role for RFS needs to be further analyzed when more data is available.

## Introduction

Curative resection followed by adjuvant chemotherapy remarkably improved survival in patients with high-risk stage II and stage III colorectal cancer (CRC). However, it has soon become clear that not all patients benefit equally and a significant proportion of patients even has worse prognosis than others. Retrospective researches and randomized clinical trials (RCTs) carried out in these patients confirmed that BRAF mutation is one of the strong negative prognostic factors [1, 2].

A negative prognostic impact of the BRAF mutation in metastatic colorectal cancer had already been corroborated by several meta-analyses [3, 4]. Irrespective of the stage of cancer, one meta-analysis has shown that mutated BRAF determines poor overall survival (OS) in colorectal cancer when compared with wild type BRAF [Hazard ratio (HR) = 2.25, 95% confidence interval (CI): 1.82–2.83] [5]. However, in the subgroup of stage II/III CRC patients treated with curative resection and adjuvant chemotherapy, the prognostic value of BRAF mutation is still uncertain. Although several RCTs including PETACC-3 [6], or NSABP C-07 and C-08 [7], recognize BRAF mutation as an unfavorable prognostic factor for OS in this subgroup of CRC, but it was not confirmed by others, take CALGB 89803 [8] and MOSAIC [9] as examples. Furthermore, in respect of disease-free survival (DFS), results from the RCTs are inconclusive: for example CALGB 89803 found no difference between BRAF-mut and BRAF-wt CRC whereas N0147 [2] observed worse DFS in the BRAF-mut subpopulation compared to the BRAF-wt subpopulation. Interestingly, the prognostic value of BRAF mutation was not the same according to different cancer sites in N0147 trial.

Therefore a systematic review and meta-analysis of RCTs was performed to quantitatively evaluate whether BRAF mutation plays a negative prognostic role in stage II/III CRC receiving curative resection and adjuvant chemotherapy.

## Methods

### Selection criteria and search strategy

Three individuals carried out the literature search. All randomized trials (1) of stage II/III CRC undergoing curative resection, followed by adjuvant chemotherapy, and (2) with sufficient quantitative data of the prognosis according to BRAF mutation status were eligible. We considered sufficient quantitative data as data including the necessary descriptive and statistical information to perform meta-analysis, such as HR, CI, observed or logrank expected events and Kaplan-Meier curve; there was no need to included all of these data, but enough data to perform meta-analysis was required. The insufficient quantitative data means the article lacked necessary information to perform meta-analysis.

This meta-analysis was performed according to the Preferred Reporting Items for Systematic Reviews and Meta-Analyses (PRISMA) statement (S1 Table) [10]. We searched PubMed, EMBASE, Web of Science and the Cochrane library using the following terms: (colon cancer or colon carcinoma or colorectal cancer or colorectal carcinoma or rectal cancer or rectal carcinoma) and (BRAF or B-RAF or B RAF) and (((stage and (II or III)) or resect* or surg* or opera* or adjuvant*). Database searches were restricted to articles published in English until 19 November 2015. Exclusion criteria were described as follows: (1) mixed prognostic data of stage I or IV with stage II/III colorectal cancer; (2) neoadjuvant chemotherapy or radiation prior to resection; (3) data partly or totally included in other eligible studies; (4) insufficient prognostic data according to BRAF mutations.

### Data extraction and quality assessment

The following data were collected from each study: author, year of publication, treatment protocol, BRAF detection, mutational data, and survival outcomes, namely, relapse-free survival (RFS), DFS and OS. Overall survival was defined from the time random assignment to the date of death, and disease free survival was calculated from the time study enrollment to tumor recurrence, occurrence of a new primary colorectal tumor, or death from any cause, while RFS was defined from the time study enrollment to tumor recurrence or occurrence of a new primary colon tumor, patients that died without these two issues were censored. Specifically, the hazard ratios and associated 95% confidence intervals of survival outcomes for BRAF-mut patients compared to those with BRAF-wt were extracted. Pooled estimates of the prognosis for survival according to the BRAF status were weighted and pooled using the generic inverse-variance. An Egger’s funnel plot was performed to access the publication bias.

### Statistical analysis

The comparative effects were initially analyzed by the traditional pairwise meta-analysis method using Cochrane Collaboration review manager software (RevMan v.5.3.0). The relative risk for dichotomous outcomes and the standardized mean difference for continuous outcomes pooled across studies were estimated by using the DerSimonian and Laird random-effects model [11]. The level of significance was set at 5%. If the result of analysis showed *p* > 0.05, the studies were considered homogeneous and a fixed-effect model for meta-analysis was chosen. Otherwise, a random-effect model was performed. Inconsistency was quantified using the *I²* statistic, *I²* <25% reflects a small level of inconsistency while *I²* >50% implies significant inconsistency. And we excluded one study each time to perform the sensitivity analysis to examine the robustness of the estimates. For all analyses, P < 0.05 was considered statistically significant.

## Results

### 1. Literature retrieval and study characteristics

**Fig 1:**
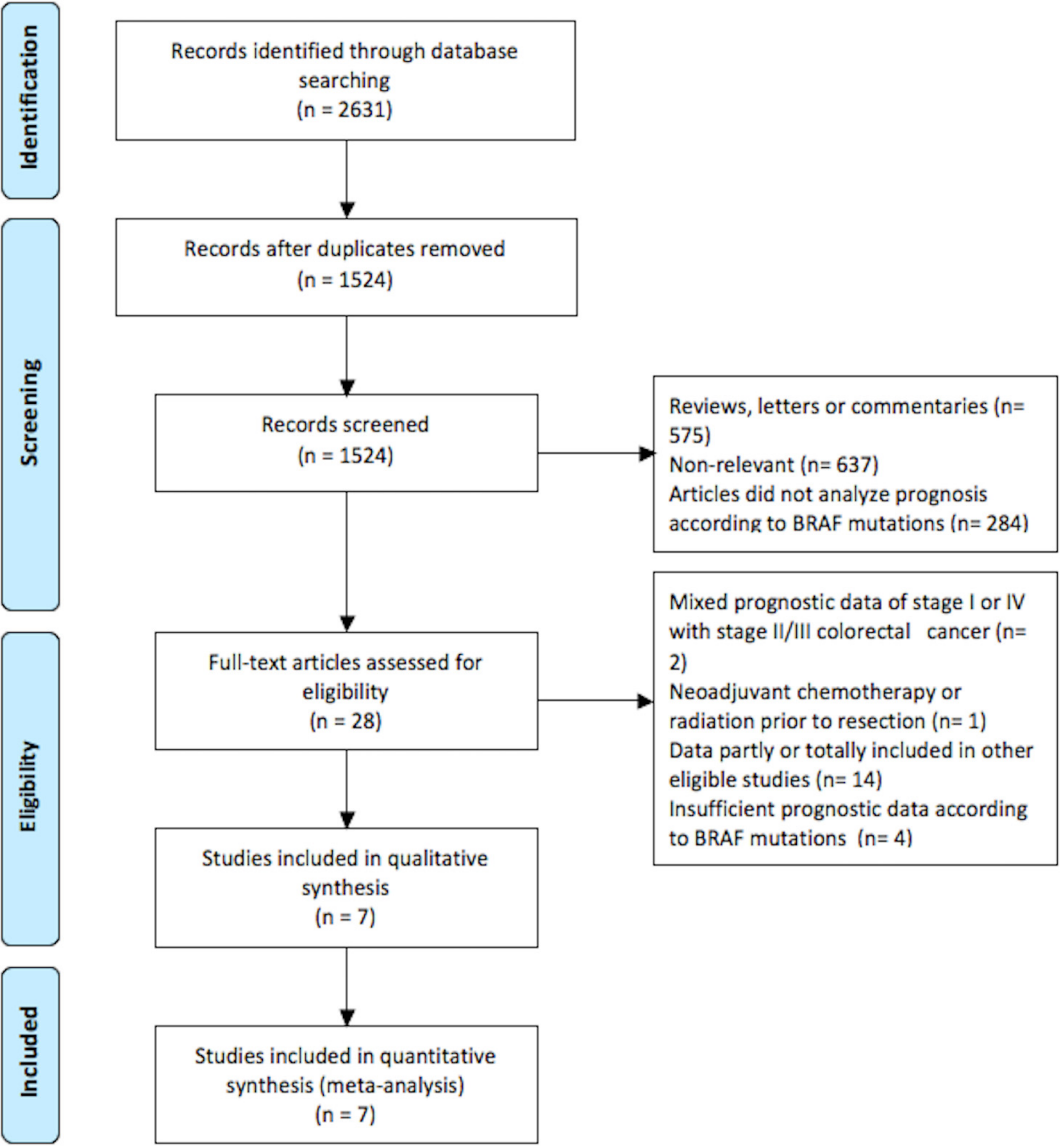
Selection of studies included in the analysis.

The literature search identified 2631 citations, of which seven phase III RCTs with 14,699 patients were considered eligible and were included in this meta-analysis. (Fig 1) Among these, 8721 patients had been assessed for the BRAF mutation, and a total of 1,035 BRAF-mut CRC patients, 11.9% of the tested population, were incorporated in these studies. Since we defined the OS, DFS and RFS as mentioned above, the RFS defined in the research of Roth et al [6] was transferred into DFS in this meta-analysis. Sinicrope et al had observed that BRAF-mut had significant negative prognostic value for DFS and OS in proximal colon cancer. However, the prognostic value of BRAF mutation was not significant for DFS and OS in colon cancer in the same report. [2] Therefore, analysis about proximal side and distal side from that study were considered as separate ones—named Sinicrope 1 (proximal colon cancer) and Sinicrope 2 (distal colon cancer)—in the meta-analysis. Although most studies included were enrolled in colon cancer, in the study of Pentheroudakis 27.4% cancers' primary site was rectum, and all the studies included both sides cancer. The characteristics and biomarker analyses of included studies are outlined in Table 1. Particularly, in these RCTs, BRAF mutations are more prominent in DNA mismatch repair (MMR) defective and microsatellite instability (MSI) high tumors, for examples, Roth et al found 46% BRAF-mut patients were with MSI-high; Gavin et al found 29% BRAF-mut patients were with dMMR, while 9% BRAF-wt patients with dMMR; in French's study [12], 45% BRAF-mut patients were with dMMR, while 6% BRAF-wt patients with dMMR, while only French's study [12] performed stratified analysis of BRAF mutation's prognostic role according to MMR status, and only Gavin et al's study [7] provided stratified analysis of MMR status' prognostic role according to BRAF mutations. Therefore, there is no sufficient data to do further stratified. None of the included studies analyzed KARS mutation's prognostic role in BRAF wild type group.

**Table 1:** Characteristics of included phase III RCTs.

**Author, year [ref]**	**Treatment**	**Total n**	**Pts with tissue available for BRAF mutations**	**Median follow-up time**	**BRAF mut type**	**BRAF assement method**	**BRAF mut n, tested % (n)**	**OS**	**DFS**	**RFS**
French, 2008 [12]	Curative surgery + FU/LV	878	490	NA	V600E	CSGE, PCR	15.7% (77)	mut VS wt HR = 1.2, 95% CI = (0.8–1.8), p = 0.31	mut VS wt HR = 1.0, 95% CI = (0.6–1.6), p = 0.97	NA
Gavin, 2012 [7]	Curative surgery + FLOX vs FU/LV, curative surgery + mFOLFOX6 vs mFOLFOX6 + Bevacizumab	4802	2226	NA	V600E, D594V,	sequenom, PCR	V600E 13.15% (293), D594V 0.80% (18)	mut VS wt HR, = 1.46; 95% CI, (1.20–1.79); P = 0.0002	NA	mut VS wt HR, 1.02; 95% CI, 0.82–1.28; P = .0.86
Roth, 2012 [6]	Curative surgery + FOLFIRI vs 5-FU/LV	2094	1404	69 months	V600E	PCR, sequencing	8% (103)	mut vs wt HR = 1.56, 95% CI = (1.02 to 2.39), p = 0 .04	mut vs wt HR = 1.17, 95% CI = (0.79 to 1.73) p = 0 .44	NA
Ogino, 2013 [8]	Curative surgery + +FU/LV vs IFL	1264	580	7.6 years	V600E	PCR, pyrosequencing	16% (94)	mut VS wt HR = 1.46, 95% CI = (1.00–2.15)	mut VS wt HR = 1.35, 95% CI = (0.94–1.93)	mut VS wt HR 1.44, CI (0.99–2.11)
Sinicrope 1, 2015 [2]	Curative surgery + mFOLFOX6 ± cetuximab	1511	1423	4.9 years	V600E	PCR, sequencing	20% (287)	mut vs wt HR = 1.58, 95% CI = (1.17–2.12),p = 0.003	mut vs wt HR = 1.35, 95% CI = (1.03–1.76),p = 0.028	NA
Sinicrope 2, 2015 [2]	Curative surgery + mFOLFOX6 ± cetuximab	1463	1375	4.9 years	V600E	PCR, sequencing	4% (54)	mut vs wt HR = 1.79, 95% CI = (0.99–3.24),p = 0.053	mut vs wt HR = 1.25, 95% CI = (0.75–2.11),p = 0.394	NA
André, 2015 [9]	Curative surgery + LV5FU2 vs FOLFOX4	2246	902	10 years	V600E	PCR, pyrosequencing	10.42% (94)	mut VS wt HR = 0.99, 95% CI = 0.67 to 1.47, p = 0.965	NA	NA
Pentheroudakis, 2015 [13]	Curative surgery + FOLFOX vs XELOX	441	321	74.7 months	V600E	PCR, sequencing	4.7% (15)	mut VS wt HR 1.75, 95% CI (0.7–4.35) p = 0.23	mut VS wt HR 1.28, 95% CI (0.52–3.17) p = 0.59	NA

PCR = polymerase chain reaction; RCT = randomized clinical trial; FOLFOX = bolus + continuous infusion 5-fluorouracil + bolus folinic acid days 1,2 + oxaliplatin; FU = 5-fluorouracil; LV = folinic acid; FLOX = bolus infusion 5-fluorouracil + bolus folinic acid + oxaliplatin; IFL = bolus irinotecan + bolus infusion 5-fluorouracil + bolus folinic acid; FOLFIRI = 5-fluorouracil + folinic acid + irinotecan; BRAF = B-Raf proto-oncogene; DFS = disease-free survival; OS = overall survival; RFS = relapse-free survival; NA = not available.

### 2. Quality of individual studies

**Fig 2:**
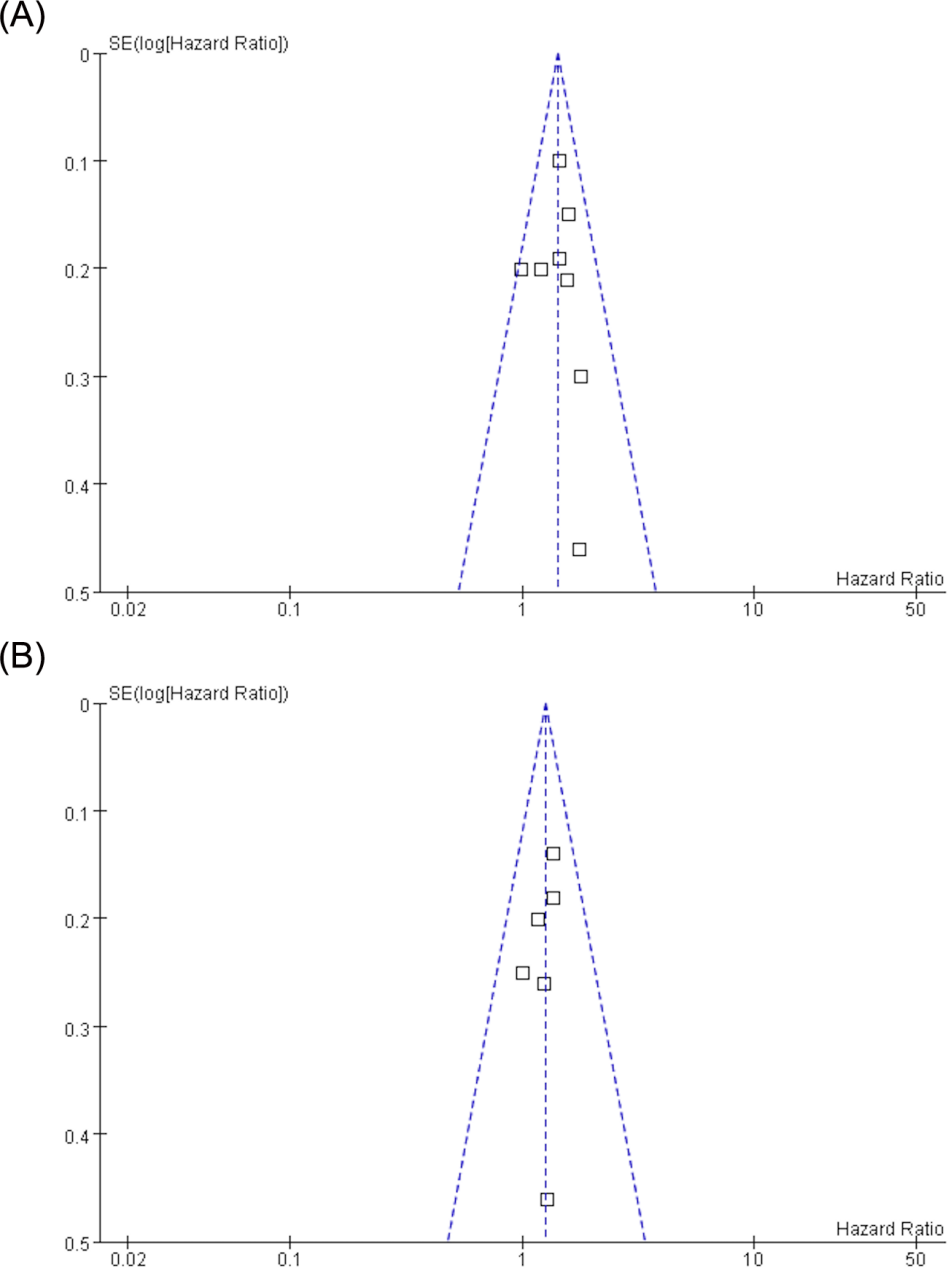
Funnel plots for publication bias of OS (a) and DFS (b) meta-analysis: no evidence of bias was found.

Among the eligible studies, seven RCTs were double blinded, and all described the randomization processes that they had used. All included a power calculation to determine the optimal sample size. The funnel plots of publication bias of OS meta-analysis provided a qualitative estimation of publication bias of the studies, and no evidence of bias was found. (Fig 2)

### 3. Prognostic role of BRAF mutation on OS in stage II/III CRC

**Fig 3:**
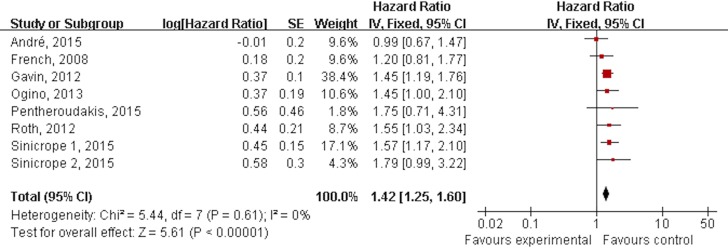
Forest plots showing hazard ratio for a negative prognostic role of BRAF mutation on over all survival in stage II/III CRC.

Among the eligible studies, eight subgroups from seven RCTs were included to assess the predictive role of BRAF mutation on OS in CRC with adjuvant chemotherapy compared to BRAF wild. No heterogeneity was found among the trials (P = 0.61, I^2^ = 0%), and a fixed-effects model was thus chosen for the analysis. The prognostic role of BRAF mutation on OS is shown in Fig 3. Based on the meta-analysis, CRC with BRAF mutation have poorer overall survival after surgery and adjuvant chemotherapy, and the HR for OS with BRAF mutation is 1.42 (95% CI, 1.25–1.60; P < 0.00001), compared to BRAF wild CRC. (Fig 3)

### 4. Prognostic role of BRAF mutation on DFS in stage II/III CRC

**Fig 4:**
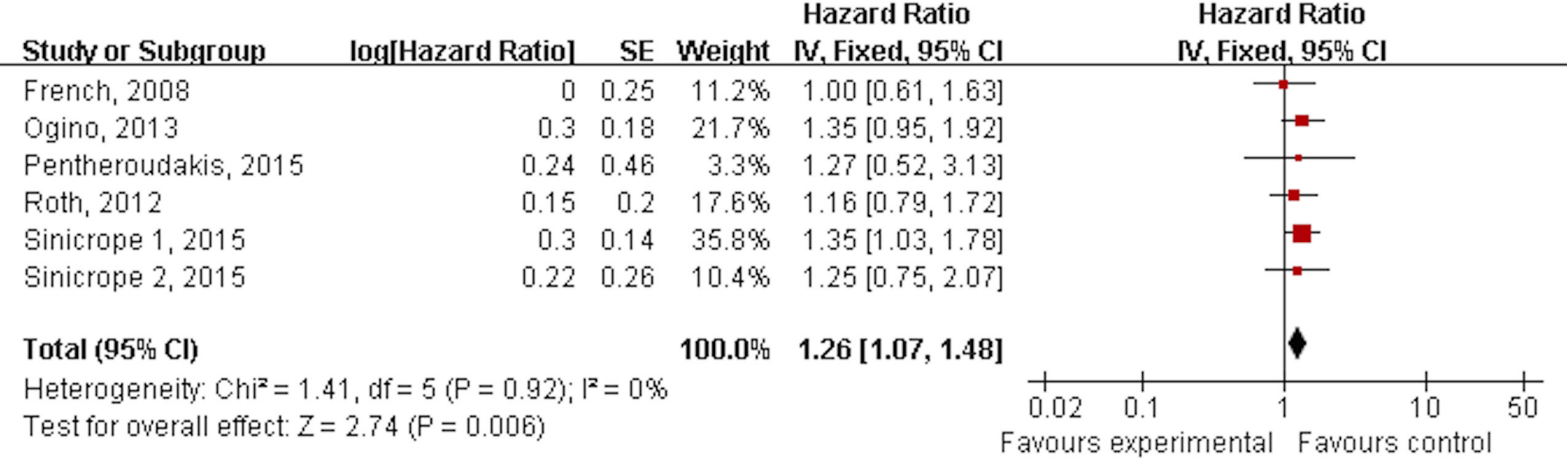
Forest plots showing hazard ratio for a negative prognostic role of BRAF mutation on disease free survival in stage II/III CRC.

There were six subgroups from five RCTs with enough data to assess the efficacy of BRAF mutation on DFS compared to BRAF wild. No heterogeneity was found among the trials (P = 0.92, I^2^ = 0%), and a fixed-effects model was thus chosen for the analysis. Based on the analysis of four RCTs, CRC with BRAF mutation had shorter DFS than those with BRAF wild (HR = 1.26, 95% CI: 1.07–1.48, P = 0.006). (Fig 4)

### 5. Prognostic role of BRAF mutation on RFS in stage II/III CRC

Among the seven RCTs, only Gavin et al [7] and Ogino et al [8] analyzed the prognostic role of BRAF mutation on RFS, reporting HRs of 1.02 (0.82–1.28) and 1.44 (0.99–2.11), respectively. Therefore, we did no not perform the meta-analysis about the prognostic role of BRAF mutation on RFS due to the limited data.

### 6. Sensitivity analysis

One study was removed at a time to conduct the sensitivity analysis. The pooled HRs and CIs were not significantly changed for OS, indicating the stability of the result. However, it is noteworthy that in the sensitivity analysis for DFS the outcome seemed similar except that when the study Sinicrope 1 excluded, the HR (95%CI) become 1.21 (0.99, 1.48), suggesting the lack of robustness of the estimate and a cautious interpretation about the result that BRAF mutation plays a unfavorable role on DFS in stage II/III CRC is needed. (Table 2)

**Table 2:** Sensitivity analysis by omitting each of the included studies in different outcomes.

**Outcomes**	**Omitted Study**	**HR (95%CI)**	**P**	**I**^**2**^	**Ph**
**OS**	André 2015	1.47 (1.29, 1.67)	< 0.00001	0	0.93
	French 2008	1.44 (1.27, 1.64)	< 0.00001	0	0.59
	Gavin 2012	1.40 (1.20, 1.63)	< 0.0001	0	0.50
	Ogino 2013	1.41 (1.24, 1.61)	< 0.00001	0	0.49
	Pentheroudakis 2015	1.41 (1.25, 1.59)	< 0.00001	0	0.52
	Roth 2012	1.40 (1.24, 1.59)	< 0.00001	0	0.52
	Sinicrope 1 2015	1.39 (1.21, 1,58)	< 0.00001	0	0.56
	Sinicrope 2 2015	1.40 (1.24, 1.59)	< 0.00001	0	0.57
**DFS**	French 2008	1.30 (1.09, 1.54)	0.004	0	0.98
	Roth 2012	1.28 (1.07, 1.53)	0.008	0	0.88
	Ogino 2013	1.23 (1.02, 1.49)	0.03	0	0.88
	Sinicrope 1 2015	1.21 (0.99, 1.48)	0.07	0	0.91
	Sinicrope 2 2015	1.26 (1.06, 1.50)	0.009	0	0.84
	Pentheroudakis 2015	1.26 (1.06, 1.49)	0.007	0	0.84

OS = overall survival; DFS = disease free survival; HR = hazard ratio; Ph = P for heterogeneity

## Discussion

The cellular RAS/RAF/Mitogen-activated protein kinase/ERK kinase (MEK)/extracellular signal regulated kinase (ERK) pathway controls cell proliferation, apoptosis and differentiation by transferring signals from cell surface receptors to transcription factors. Activated by RAS, the RAF phosphorylates and activates downstream MEK, which then activates ERK subsequently and ultimately results in the activation of transcription factors. Mutations of these signals can cause abnormal cell growth, invasion and metastasis. Among these, RAF consists of serine–threonine kinases ARAF, BRAF and CRAF. While ARAF and CRAF mutations are detected only rarely, BRAF mutations are found at a higher frequency in several cancers. Meanwhile, 90% BRAF mutations are V600E mutation, which is reported to occur in melanoma (40–60%), papillary thyroid carcinoma (45%), low grade serous ovarian carcinoma (35%), colorectal adenocarcinoma (5–15%) and other cancers [14]. Besides, Zheng et al found that about 20.6% of BRAF-mut tumors in CRC are non-V600E mutations, including N581S, Y472C, D594G and D594N mutations, and the latter two are related to concomitant activation of KRAS or NRAS mutations [15]. Patients harboring BRAF 594 or 596 mutated CRC have a longer OS than BRAF wild type and BRAF V600E mutated CRC (62.0 versus 35.9 months versus 12.6 months; HR = 0.55, P = 0.081 and HR 0.36, P = 0.002) [16].

In CRC, while it is nearly mutually exclusive with KRAS mutation, BRAF mutation is associated with specific high-risk clinicopathologic characteristics, such as lymph-node metastasis, high grade, T4 tumors, mucinous histology, MSI or MMR and right-side location [8, 9]. Recently, Loupakis et al found that CRC patients with right-side primary, female gender and mucinous histology have 81% chance to bear a BRAF V600E-mutant [17, 18]. Since these characteristics themselves are related to worse survival, it may be possible that the unfavorable prognostic value of BRAF mutation and these high-risk factors are interactive. For example, BRAF V600E mutation is detected in less than 12% MMR-proficient tumors, while in MMR-defective tumors the incidence ranges from 35 to 60% [7, 12]. It was reported that the prognostic effect of BRAF and MMR status is additive, giving rise to worst prognosis for patients with MMR-proficient and BRAF-mutant tumors [7]. Similar results were observed in patients according to MSI or microsatellite stability (MSS). On the other hand, compared to the right-sided BRAF-mut CRC, the left-sided BRAF-mut CRC had a worse prognosis. Hence, the MSS/left side BRAF-mut population had the worst survival [17]. However, it should be reminded that these clinical and pathologic features are typically all bundled together.

Several meta-analyses have demonstrated that advanced CRC with BRAF-mut has a much poorer prognosis compared to those with BRAF-wt [19, 20]. Conversely, the prognostic role of BRAF mutation has not been clarified in stage II and stage III CRC patients undergoing adjuvant chemotherapy after curative resection. The actual meta-analysis that quantitatively and systematically collected the evidence from seven RCTs is the first one to demonstrate that the presence of BRAF mutation plays a negative prognostic role for OS in the patients. As for DFS, in the sensitivity analysis when the study Sinicrope 1 was removed, the unfavorable prognostic value of BRAF mutation was not statistically confirmed, with HR = 1.21, 95% CI: 0.99–1.48, P = 0.07, indicating the instability of the result. Hence, whether BRAF mutation is related with shorter DFS in stage II/III CRC needs a more cautious interpretation with the following two facts: 1) among the six studies included in the analysis about BRAF mutation's prognostic value on DFS, the study Sinicrope 1 contains the largest number of BRAF-mut patients and has the narrowest 95% confidence interval (1.03–1.76), therefore the result of this study affect the meta-analysis' outcome remarkably, and deleting it could change the result significantly. 2) all patients in the study Sinicrope 1 are proximal colon cancer, which not only is associated with high prevalence of BRAF mutation, but also, more importantly, indicates poor prognosis itself. The proximal colon location and BRAF mutation can synergistically play adverse role on the prognosis, and these patients may have the shorter DFS, thus the DFS may be better after removing the study Sinicrope 1. Nevertheless, when the study was removed, *I*^*2*^ of the heterogeneity remained to be 0%, meaning that the results of this meta-analysis is reliable in terms of the whole BRAF mutation population regardless of cancer locations. Consequently, the BRAF mutation has a unfavorable prognostic value on DFS in stage II/III CRC without regard to cancer locations and other situations. Certainly, it would be better to perform stratified analysis based on cancer locations to check whether BRAF mutation in proximal colon cancer is confirmed a adverse prognostic factor, while its prognostic value is not sure in distal colon cancer. Unfortunately we cannot work it out since only the study Sinicrope performed stratified analysis of BRAF mutation's prognostic role according to cancer locations. Furthermore, a higher hazard ratio for OS than DFS in BRAF-mut patients may imply that population has a shorter survival time after recurrence in comparison to BRAF-wt patients, as suggested by Gavin et al [7].

Despite its negative prognostic value confirmed in this meta-analysis, there is no evidence of a predictive value of BRAF mutation in the terms of therapy outcomes. In stage II/III CRC, BRAF-mut patients receiving adjuvant chemotherapy plus cetuximab had no survival advantage over those receiving adjuvant chemotherapy only [21]. Moreover, the study of Ogino et al suggested no significant predictive value of BRAF mutation for irinotecan-based adjuvant chemotherapy either [22]. Similarly, meta-analyses have neither demonstrated statistical differences of anti-EGFR monoclonal antibodies' (mAbs) effect on progressive-free survival (PFS) or OS between BRAF-mut and BRAF-wt metastatic colorectal cancer, nor benefit from the addition of anti-EGFR mAbs to standard chemotherapy in BRAF-mut patients compared with chemotherapy alone or best support care [23, 24]. Fortunately, compared to FOLFIRI plus bevacizumab, FOLFOXIRI plus bevacizumab seems to generate better PFS and OS in BRAF-mut advanced CRC [25].

The following issues should also be taken into account when interpreting the results of this meta-analysis. First, because of the relatively low prevalence of BRAF mutation in CRC, the proportion of BRAF-mut patients is limited. Moreover, even though all the prognostic data were derived from phase III randomized clinical trials, some studies included in the meta-analysis analyzed BRAF status retrospectively, leading to about 6–60% of patients lacking information about BRAF mutational status causing that it might miss a positive prognostic value during the evaluation. Second, since this analysis has been performed based on published and not on individual patient data, specific subgroup analyses could not be conducted, such as further stratified analysis according to MSI/MMR status in BRAF-mut patients. Third, the chemotherapies received in some trials are not used as standard management in stage II or III colorectal cancer. Besides, two RCTs contain irinotecan as treatment while five contain oxaliplatin, and bevacizumab is included in one study include compared to cetuximab in two study. However it is infeasible to do further stratified according to the regimens since these studies mixed the survival data from different therapies together. Finally, due to the uncommonness of most BRAF mutation types, this meta-analysis almost exclusively evaluated the prognostic value of the BRAF V600E mutation. For example, about 0.80% (n = 18) BRAF mutation in NSABP C-07 and C-08 clinical trials were BRAF D594G mutations, but such rare or undetected BRAF mutation types may not inevitably carry a similar prognostic value as BRAF V600E mutation. Nonetheless, this study is based on the most exhaustive overview of traceable randomized trials conducted so far in stage II/III CRC with curative resection and adjuvant chemotherapy. Furthermore, this is the first meta-analysis that suggests a negative prognostic role of the BRAF V600E mutation for this group of CRC patients.

In summary, this meta-analysis shows that BRAF mutation is significantly related with worse DFS and OS among stage II/III CRC patients receiving adjuvant chemotherapy after curative resection, while its prognostic role for RFS needs to be further analyzed when more data is available. Future studies, especially well-designed large randomized controlled trials conducted in BRAF-mut stage II/III CRC patients are urgently needed to find better treatments for that population.

## Supporting Information

S1 Table PRISMA checklist. (DOC)
